# The Acceptance and Use of Digital Technologies for Self-Reporting Medication Safety Events After Care Transitions to Home in Patients With Cancer: Survey Study

**DOI:** 10.2196/47685

**Published:** 2024-03-08

**Authors:** Yun Jiang, Misun Hwang, Youmin Cho, Christopher R Friese, Sarah T Hawley, Milisa Manojlovich, John C Krauss, Yang Gong

**Affiliations:** 1 School of Nursing University of Michigan Ann Arbor, MI United States; 2 Rogel Cancer Center University of Michigan Ann Arbor, MI United States; 3 McWilliams School of Biomedical Informatics The University of Texas Health Science Center at Houston Houston, TX United States; 4 School of Public Health University of Michigan Ann Arbor, MI United States; 5 Department of Internal Medicine University of Michigan Ann Arbor, MI United States; 6 VA Ann Arbor Center for Clinical Management Research Ann Arbor, MI United States

**Keywords:** digital technology, patient safety, patient participation, patient-reported outcomes, drug-related side effects and adverse reactions

## Abstract

**Background:**

Actively engaging patients with cancer and their families in monitoring and reporting medication safety events during care transitions is indispensable for achieving optimal patient safety outcomes. However, existing patient self-reporting systems often cannot address patients’ various experiences and concerns regarding medication safety over time. In addition, these systems are usually not designed for patients’ just-in-time reporting. There is a significant knowledge gap in understanding the nature, scope, and causes of medication safety events after patients’ transition back home because of a lack of patient engagement in self-monitoring and reporting of safety events. The challenges for patients with cancer in adopting digital technologies and engaging in self-reporting medication safety events during transitions of care have not been fully understood.

**Objective:**

We aim to assess oncology patients’ perceptions of medication and communication safety during care transitions and their willingness to use digital technologies for self-reporting medication safety events and to identify factors associated with their technology acceptance.

**Methods:**

A cross-sectional survey study was conducted with adult patients with breast, prostate, lung, or colorectal cancer (N=204) who had experienced care transitions from hospitals or clinics to home in the past 1 year. Surveys were conducted via phone, the internet, or email between December 2021 and August 2022. Participants’ perceptions of medication and communication safety and perceived usefulness, ease of use, attitude toward use, and intention to use a technology system to report their medication safety events from home were assessed as outcomes. Potential personal, clinical, and psychosocial factors were analyzed for their associations with participants’ technology acceptance through bivariate correlation analyses and multiple logistic regressions.

**Results:**

Participants reported strong perceptions of medication and communication safety, positively correlated with medication self-management ability and patient activation. Although most participants perceived a medication safety self-reporting system as useful (158/204, 77.5%) and easy to use (157/204, 77%), had a positive attitude toward use (162/204, 79.4%), and were willing to use such a system (129/204, 63.2%), their technology acceptance was associated with their activation levels (odds ratio [OR] 1.83, 95% CI 1.12-2.98), their perceptions of communication safety (OR 1.64, 95% CI 1.08-2.47), and whether they could receive feedback after self-reporting (OR 3.27, 95% CI 1.37-7.78).

**Conclusions:**

In general, oncology patients were willing to use digital technologies to report their medication events after care transitions back home because of their high concerns regarding medication safety. As informed and activated patients are more likely to have the knowledge and capability to initiate and engage in self-reporting, developing a patient-centered reporting system to empower patients and their families and facilitate safety health communications will help oncology patients in addressing their medication safety concerns, meeting their care needs, and holding promise to improve the quality of cancer care.

## Introduction

### Background

The rapid growth in cancer treatment options has contributed to improved survival but has increased the complexity of care [1]. Most adults with cancer now receive their treatments in outpatient settings, and an increasing number of patients take cancer medications orally in their homes [2]. This shift in cancer care increases the likelihood of transitions across diverse settings, including primary care facilities, cancer centers, community infusion clinics, and homes [3]. Frequent care transitions may lead to medication safety events owing to inaccurate medication information sharing or poor communication [4]. Patients often face challenges in managing complex dosage schedules of their cancer medications, potentially life-threatening toxicities, and highly incident drug-drug and drug-food interactions at home [5]. Furthermore, many patients with cancer take concomitant medications for other chronic conditions [6]. During care transitions, these medications may be stopped, started, or changed, and unintentional changes at these interfaces can lead to discrepancies, which may, in turn, lead to adverse medication events. Currently, there is a lack of complete understanding of the nature, scope, and causes of medication safety events that patients with cancer experience at home because of a lack of patient engagement in self-reporting safety events from home [7].

Active engagement of patients with cancer and their families in self-monitoring and reporting adverse medication events from home is indispensable to achieving safe and effective care transitions and optimal patient outcomes [8]. However, patient and family engagement has been limited, especially in oncology settings [9-11]. There are numerous barriers to such engagement, including knowledge, attitudes and beliefs, health literacy, cultural differences, sex, age, education, economic status, and disease and symptom burdens [8,12]. Moreover, patients may be unable to engage when they receive conflicting recommendations or are excluded from the care-planning process [8]. As a result, patients may withhold their concerns regarding medication safety, be unwilling or unable to report to clinicians, or even fear reprisals from clinicians [7,13].

A patient-centered medication safety self-reporting system can guide and engage patients with cancer in self-management and reporting their experiences and concerns regarding medication events. The Chronic Care Model (CCM) highlights digital technologies’ support for productive communications between informed, activated patients and a well-prepared, proactive practice team to improve outcomes [14]. Furthermore, emerging evidence demonstrates the effectiveness of patient-facing technology solutions to empower the patient’s well-being and help strengthen the relationship and communication between patients with cancer and their health care providers [15,16]. However, there are challenges in initiating and engaging people with cancer using technology systems for health self-monitoring and health communications [17-19]. A 2017 pilot study of a web- and telephone-based safety reporting system received only 37 reports in 17 months [20]. Lessons from this pilot project include increased patient engagement and the focus on high-risk and high-reward populations at risk for notable adverse events [20,21], which are particularly applicable to patients with cancer during care transitions. However, the challenges faced by patients with cancer in adopting digital technologies and engaging in medication safety event self-reporting during transitions of care have not been fully understood.

Existing electronic patient-reported outcome systems for patients with cancer are often limited to reporting preselected common symptoms using survey questionnaires, which are not able to address patients’ various experiences and concerns regarding medication safety during care transitions. In addition, these systems are not designed for patients to initiate timely self-reporting [21-23]. The willingness of oncology patients to use digital technologies to report medication safety events from home settings has been less studied [23,24]. The literature from other clinical settings suggests 3 prerequisites regarding the psychosocial aspects for patient willingness to share safety concerns: cognitive-cultural conditions (eg, patients’ understanding and prioritization of patient safety); structural-procedural conditions (eg, the opportunity, means, and ease of providing feedback); and learning and change conditions (eg, feeling that their feedback would be acted upon and make a difference to patient safety) [7]. These prerequisite conditions can be further explored among patients with cancer to identify factors associated with their willingness to engage in medication safety event self-reporting after their care transitions to home.

### Objectives

In this survey study, we aimed to assess oncology patients’ perceptions of medication and communication safety during care transitions and their willingness to use digital technologies to self-report medication safety events after care transitions to home and to identify factors associated with their technology acceptance. This study’s findings support subsequent development and testing of personalized technology systems for patients with cancer to self-report medication safety events to improve patient-centered cancer care, especially during care transitions.

## Methods

### Study Design and Participants

A cross-sectional survey study was conducted with patients with cancer who had received care at the University of Michigan Rogel Cancer Center, a National Cancer Institute–designated Comprehensive Cancer Center in the Midwest, from December 10, 2021, to August 30, 2022. This survey study was conducted following the STROBE (Strengthening the Reporting of Observational Studies in Epidemiology) guidelines [25] (Multimedia Appendix 1). Eligible participants were screened from electronic health records based on the following inclusion criteria: (1) receiving a diagnosis of invasive colorectal, lung, breast, or prostate cancer; (2) being discharged from the hospital or clinic to a home setting in the past 1 year; and (3) being aged ≥18 years. The 4 cancer types highlight the diversity and representation of participants, and the initial eligibility screening was determined based on the *International Classification of Diseases, Tenth Revision* codes. Multimedia Appendix 2 lists the corresponding *International Classification of Disease, Tenth Revision* codes for the 4 types of cancer.

### Recruitment and Survey Administration

**Figure 1:**
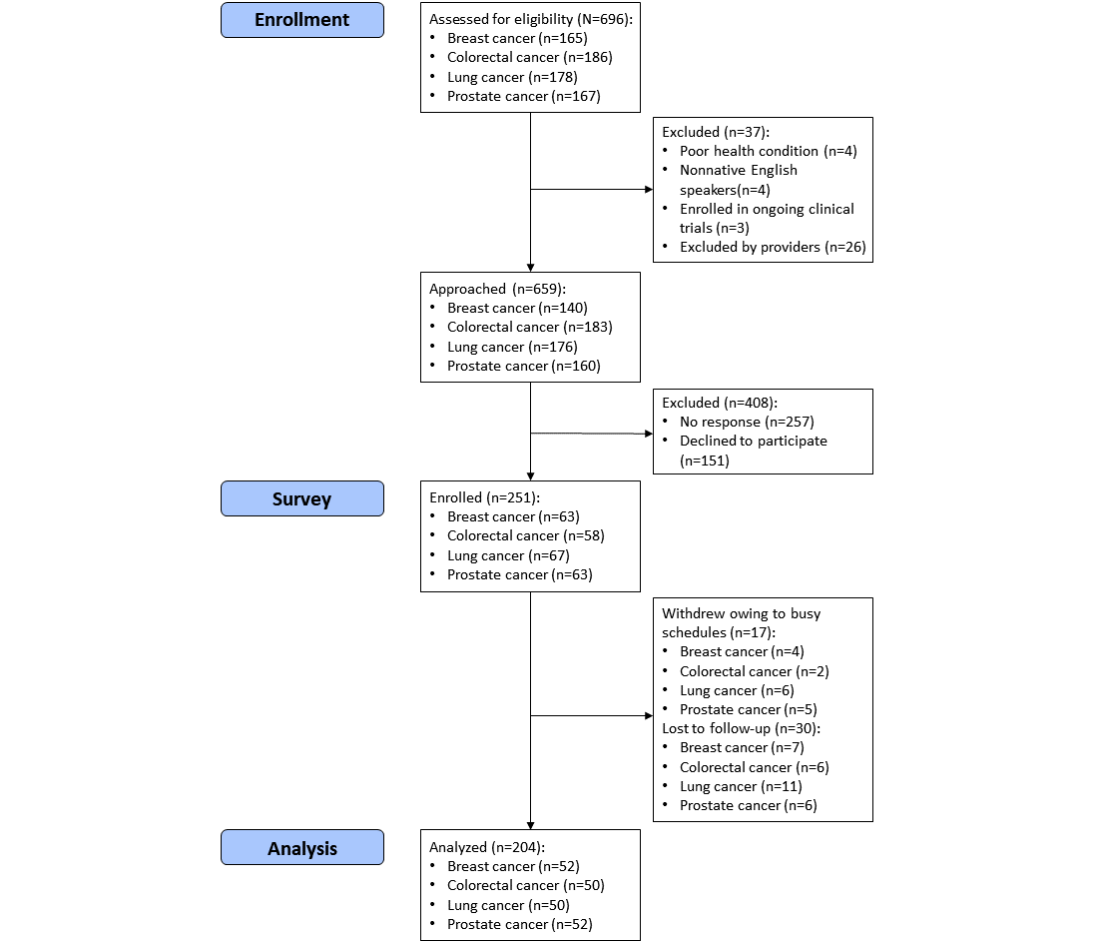
Flowchart of survey participant recruitment and enrollment process.

Figure 1 shows the survey participant recruitment and enrollment process. We recruited participants through convenience sampling. A total of 696 patients were initially identified from the medical record review, of whom 11 (1.6%) were excluded for the following reasons: poor health conditions (4/696, 0.6%), non–English speaker (4/696, 0.6%), or enrolled in an ongoing clinical trial (3/696, 0.4%). An additional 26 (3.7%) patients were excluded at their health care provider’s discretion, resulting in 659 (94.7%) patients who were contacted via either a phone call or an email invitation. Of these 659 patients, 251 (38.1%) expressed their willingness to participate in the survey. Of the 251 participants, 17 (6.8%) withdrew from the study owing to their busy schedules and 30 (11.9%) were lost to follow-up. A total of 204 (81.3%) participants completed the surveys, including 181 (88.7%) participants who submitted using the web-based Qualtrics survey platform, 55 (26.9%) via email, and 9 (4.4%) via phone call.

### Ethical Considerations

This study was determined to be an exempt study by the University of Michigan Institutional Review Board (HUM00203239). Informed consent was obtained from all participants before data collection via phone, email, or the internet. Anonymized survey data were used for analysis. The respondents who completed the survey were given a US $25 gift card to appreciate their time and effort.

### Measures

The survey included 66 items that were generated by the research team or adapted from the literature. Participants’ acceptance of using digital technologies for self-reporting medication safety events from home as the primary outcome was assessed after a short scenario that described the functionalities of a web-based medication safety event self-reporting system and how it worked. In total, 4 questions were asked about participants’ (1) perceived usefulness (ie, “How likely would you consider this online reporting tool is useful?”), (2) perceived ease of use (“How likely would you consider it is easy to use such an online reporting tool?”), (3) attitudes toward use (“How would you think about reporting your safety experiences or concerns through such an online reporting tool?”), and (4) intention to use this self-reporting system (“Do you intend to use an online reporting tool to report your safety concerns?”), using a 5-point Likert scale, ranging from “very unlikely” to “very likely.” A total of 3 prerequisites, including cognitive-cultural conditions (eg, medication safety perception, perceived safety of communication with physicians, self-rated health, patient activation, and medication self-management ability); structural-procedural conditions (eg, experience with web-based self-reporting systems); and learning and change conditions (eg, perceived importance of feedback and influence of others’ self-reporting responses), were assessed as potential psychosocial factors. Specifically, medication safety perceptions (4 items), perceived safety of communication with a physician (2 items) [7], self-rated health status (4 items), beliefs about medications (10 items) [26], patient activation (13 items) [27], and medication self-management ability (10 items) [28] were assessed as cognitive-cultural conditions. Prior technology use experiences (6 items), including prior experience with web-based health-related information self-reporting systems (1 item), were assessed as the structural-procedural conditions. Learning and change condition measures included the perceived importance of feedback on their reports (3 items) and the perceived influence of seeing others’ self-reporting responses (1 item). Furthermore, we administered a brief sociodemographic questionnaire (9 items) to collect personal information, such as age, sex, race, ethnicity, income, education, marital status, and employment status. We extracted clinical factors from patients’ electronic medical records, including cancer types and whether they were taking oral anticancer agents (OAAs) and other outpatient medications. The participants were informed that they could decline to answer any question that they preferred not to respond to, and they also had the choice to stop the survey at any point. Refer to Multimedia Appendix 3 for the survey questionnaires.

### Statistical Analysis

The characteristics of participants were summarized using descriptive statistics (ie, mean, SD, frequency, and percentage). The associations between each potential factor and participants’ safety perceptions and acceptance of the medication safety self-reporting system were assessed using bivariate correlation analyses. Multiple logistic regression was conducted to identify the adjusted associations between factors and each technology acceptance variable (ie, perceived usefulness, perceived ease of use, attitude toward use, and intention to use, recoded as “likely” vs “unlikely or uncertain”) after controlling for all personal and clinical characteristics and selected psychosocial factors that presented *P*<.20 in bivariate analyses. Two-sided *P* values of ≤.05 were considered statistically significant. All statistical analyses were conducted using Stata SE software (version 17.0; StataCorp), and the correlation matrix figure was generated by R software (version 4.2.2; R Foundation for Statistical Computing). A power analysis was conducted to justify a sample size of 204 participants, which was deemed sufficient to identify the estimated squared multiple correlation coefficient of 0.35 through multiple logistic regression using the method introduced by Hsieh et al [29].

## Results

### Summary of Sample Characteristics and Covariate Factors

#### Personal and Clinical Factors

Table 1 provides a summary of participants’ personal and clinical characteristics. The mean age of participants was 65.2 (SD 11) years. The sample had a slightly higher number of female participants (108/204, 52.9%), a majority of whom were White participants (179/204, 87.7%), college educated or above (108/204, 52.9%), currently married or living as married (153/204, 75%), and without a full-time or part-time job (150/204, 73.5%). The diagnosis of lung, breast, prostate, and colorectal cancer was approximately equally distributed among the participants. Most participants were taking OAAs currently or previously (140/204, 68.6%) and taking other outpatient medications (146/204, 71.6%) currently.

**Table 1:** Summary of personal and clinical factors of the study participants (N=204).

Characteristics		Participants
Age (y), mean (SD)		65.2 (11.0)
**Sex, n (%)**		
	Male	96 (47.1)
	Female	108 (52.9)
**Race, n (%)**		
	Racial and ethnic minority individuals^a^	25 (12.3)
	White^a^	179 (87.7)
**Marital status, n (%)**		
	Currently married or living as married	153 (75)
	Currently unmarried	51 (25)
**Educational background, n (%)**		
	College graduate or postgraduate	108 (52.9)
	Grade school, high school, or some college	96 (47.1)
**Occupation, n (%)**		
	Unemployed, retired, disabled, or others	150 (73.5)
	Full-time or part-time employee	54 (26.5)
**Cancer type, n (%)**		
	Colorectal cancer	50 (24.5)
	Prostate cancer	52 (25.5)
	Lung cancer	50 (24.5)
	Breast cancer	52 (25.5)
**Taking OAAs^b^ currently or previously, n (%)**		
	Yes	140 (68.6)
	No	64 (31.4)
**Taking other outpatient medications currently, n (%)**		
	Yes	146 (71.6)
	No	58 (28.4)

^a^Racial and ethnic minority individuals include Black or African American, Asian, or those who identified with >1 race.

^b^OAA: oral anticancer agent.

#### Psychosocial Factors

Table 2 provides a summary of participants’ psychosocial factors represented as 3 conditions: cognitive-cultural conditions (eg, medication safety perception, perceived safety of communication with physicians, self-rated health, patient activation, and medication self-management ability); structural-procedural conditions (eg, experience of web-based self-reporting systems); and learning and change conditions (eg, perceived importance of feedback and influence of others’ self-reporting responses).

**Table 2:** Summary of psychosocial factors of the study participants (N=204).

Categories			Participants
Medication safety perception, mean (SD)^a^			4.4 (0.48)
Perceived safety of communication with a physician, mean (SD)^a^			4.5 (0.59)
**Self-rated health, n (%)**			
	Good, very good, or excellent	131 (64.2)	
	Poor or fair	73 (35.8)	
**Patient activation level, n (%)**			
	1 (disengaged and overwhelmed)	15 (7.4)	
	2 (becoming aware but still struggling)	50 (24.5)	
	3 (taking action and gaining control)	92 (45.1)	
	4 (maintaining behaviors and pushing further)	47 (23)	
Medication self-management, mean (SD)^b^			9.1 (2.0)
**Medication self-management ability, n (%)**			
	≥10 (adequate)	95 (46.6)	
	<10 (inadequate)	109 (53.4)	
**Experience of web-based self-reporting systems, n (%)**			
	Yes	76 (37.3)	
	No	128 (62.7)	
**Perceived importance of feedback, n (%)**			
	Very important	161 (78.9)	
	Others	43 (21.1)	
**Influence of others’ self-reporting responses, n (%)**			
	A lot or little	103 (50.5)	
	Not at all or do not know	101 (49.5)	

^a^Scores range from 1 to 5, with higher scores indicating higher safety perception.

^b^Scores range from 0 to 12, with higher scores indicating a higher level of medication self-management.

Regarding cognitive-cultural conditions, participants reported strong perceptions of medication safety (mean 4.4, SD 0.48) and perceived safety of communication with a physician (mean 4.5, SD 0.59). More than half of the participants (131/204, 64.2%) reported good or better self-rated health, at least 68.1% (139/204) reported level 3 patient activation (ie, being able to take action to maintain and improve health), and approximately 46.6% (95/204) had adequate medication self-management ability. Regarding structural-procedural conditions, more than one-third of participants (76/204, 37.3%) had prior experience using web-based self-reporting systems. As for learning and change conditions, most participants (161/204, 78.9%) perceived receiving feedback on their self-reporting as very important, and approximately half of the participants (103/204, 50.5%) considered that seeing other people’s self-reporting responses would influence their self-reporting.

#### Correlations Among Factors

**Figure 2:**
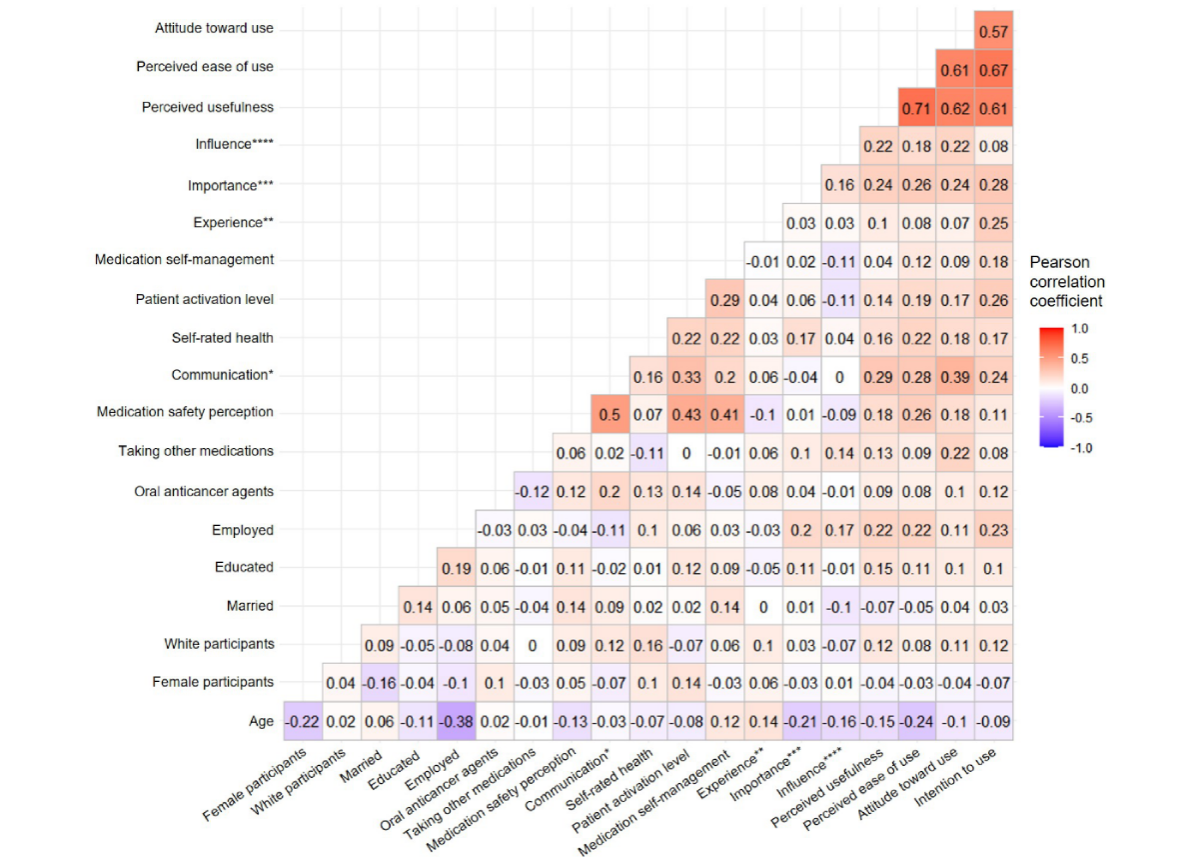
Correlation coefficient between variables. *Perceived safety of communication with a physician; **experience of web-based reporting system; ***perceived importance of feedback; ****influence of other’s reporting responses.

Figure 2 shows the results of bivariate correlation analyses between variables. Specifically, perceived medication safety and perceived safety of communication with a physician were moderately correlated with each other (*r*=0.5). Patient activation level (*r*=0.43) and medication self-management ability (*r*=0.41) were positively correlated with perceived medication safety and perceived safety of communication with a physician (*r*=0.33 and *r*=0.2, respectively).

### Summary of Technology Acceptance

As presented in Table 3, most participants perceived a medication safety event self-reporting system as useful (158/204, 77.5%) and easy to use (157/204, 77%). The majority (162/204, 79.4%) reported positive attitudes toward use. More than half of the participants (129/204, 63.2%) were willing to use the self-reporting system to report medication safety events or concerns.

**Table 3:** Summary of technology acceptance (N=204).

Categories			Participants, n (%)
**Perceived usefulness**			
	Very likely or likely	158 (77.5)	
	Very unlikely, unlikely, or uncertain	46 (22.5)	
**Perceived ease of use**			
	Very likely or likely	157 (77)	
	Very unlikely, unlikely, or uncertain	47 (23)	
**Attitude toward using**			
	Positive	162 (79.4)	
	Negative or neither	42 (20.6)	
**Intention to use**			
	Very likely or likely	129 (63.2)	
	Very unlikely, unlikely, or uncertain	75 (36.8)	

### Factors Associated With Technology Acceptance

#### Overview

Bivariate analyses indicated that several factors, including perceived safety of communication with a physician, self-rated health, patient activation, and perceived importance of receiving feedback, were significantly correlated with all technology acceptance variables, including perceived usefulness, perceived ease of use, attitude toward use, and intention to use (correlation coefficients ranged from *r*=0.14 to *r*=0.39). Medication self-management ability (*r*=0.18) and prior experience with web-based self-reporting systems (*r*=0.25) were correlated with intention to use only. Medication safety perception and the influence of seeing others’ self-reporting responses were significantly correlated with perceived usefulness, perceived ease of use, and attitude toward use (correlation coefficients ranged from *r*=0.18 to *r*=0.26) but not with the intention to use. Among other personal or clinical factors, age was negatively associated with perceived usefulness and ease of use (*r*=−0.15 and *r*=−0.24, respectively), and taking other outpatient medications was associated with a positive attitude toward use (*r*=0.22). All technology acceptance and intention to use variables were strongly correlated with each other, with coefficients ranging from *r*=0.57 to *r*=0.71 (Figure 2).

#### Perceived Usefulness

Logistic regression modeling indicated that the perceived safety of communication with a physician (odds ratio [OR] 3.59, 95% CI 1.52-8.48), the importance of receiving their self-reporting feedback (OR 2.59, 95% CI 1.03-6.51), and the influence of viewing others’ self-reporting responses (OR 2.53, 95% CI 1.08-5.91) were independent predictors of the perceived usefulness of the web-based self-reporting system, after controlling for other variables in the model. In addition, the odds of perceived usefulness were 73% lower among participants who were married or those who were living as married (OR 0.27, 95% CI 0.09-0.81), higher among those with a college graduate or postgraduate educational background (OR 2.55, 95% CI 1.06-6.12), and higher among those with a full-time or part-time occupation (OR 6.64, 95% CI 1.65-26.79). Participants’ clinical factors were not associated with the perceived usefulness of the web-based self-reporting system (Table 3).

#### Perceived Ease of Use

Logistic regression modeling indicated that the perceived medication safety (OR 3.37, 95% CI 1.07-10.58) and safety of communication with a physician (OR 2.44, 95% CI 1.05-5.68) were independent predictors of perceived ease of use of the web-based self-reporting system, after controlling for other variables in the model. In addition, the odds of perceived ease of use were 4 times higher among patients with a full-time or part-time occupation than among those without an occupation (OR 4, 95% CI 1.05-15.17). The participants’ clinical factors were not associated with the perceived ease of use of the web-based self-reporting system (Table 3).

#### Attitude Toward Use

Logistic regression modeling indicated that the perceived safety of communication with a physician (OR 9.06, 95% CI 2.95-27.84), the importance of receiving their self-reporting feedback (OR 2.99, 95% CI 1.11-8.05), and the influence of viewing others’ self-reporting responses (OR 3.36, 95% CI 1.3-8.68) were independent predictors of attitude toward use, after controlling for other variables in the model. In addition, the odds of having a positive attitude toward use were 4.23 times higher among patients taking outpatient medications (OR 4.23, 95% CI 1.63-10.99). Participants’ personal factors were not associated with attitude toward use (Table 3).

#### Intention to Use

Logistic regression modeling indicated that the perceived safety of communication with a physician (OR 2.68; 95% CI 1.17-6.11), patient activation (OR 1.83, 95% CI 1.12-2.98), previous experience with the web-based self-reporting system (OR 3.8, 95% CI 1.73-8.36), and perceived importance of receiving their self-reporting feedback (OR 3.27, 95% CI 1.37-7.78) were independent predictors of intention to use the web-based self-reporting system, after controlling for other variables in the model. In addition, the odds of intention to use were 3.4 times higher among patients with a full-time or part-time occupation than among those without an occupation (OR 3.4, 95% CI 1.26-9.19). Participants’ clinical factors were not associated with intention to use the web-based self-reporting system (Table 4).

**Table 4:** Factors associated with technology acceptance.

Factors		Perceived usefulness, OR^a^ (95% CI)	Perceived ease of use, OR (95% CI)	Attitude toward use, OR (95% CI)	Intention to use, OR (95% CI)
Age (y)		1.01 (0.96-1.06)	0.97 (0.92-1.02)	1.01 (0.97-1.07)	0.99 (0.95-1.02)
**Sex**					
	Male	Reference	Reference	Reference	Reference
	Female	0.8 (0.32-1.96)	0.58 (0.23-1.43)	1 (0.38-2.63)	0.62 (0.28-1.37)
**Race**					
	Racial and ethnic minority individuals	Reference	Reference	Reference	Reference
	White	3.29 (0.96-11.28)	1.9 (0.53-6.8)	2.25 (0.61-8.25)	2.32 (0.79-6.77)
**Marital status**					
	Currently unmarried	Reference	Reference	Reference	Reference
	Currently married or living as married	*0.27 (0.09-0.81)* ^b^	0.34 (0.12-1)	0.86 (0.29-2.54)	0.81 (0.34-1.88)
**Educational background**					
	Grade school, high school, or some college	Reference	Reference	Reference	Reference
	College graduate or postgraduate	*2.55 (1.06-6.12)*	1.56 (0.67-3.63)	1.89 (0.75-4.8)	1.22 (0.59-2.51)
**Occupation**					
	Unemployed, retired, disabled, or others	Reference	Reference	Reference	Reference
	Full-time or part-time employee	*6.64 (1.65-26.79)*	*4 (1.05-15.17)*	2.24 (0.64-7.84)	*3.4 (1.26-9.19)*
**Taking OAAs^c^ currently or previously**					
	No	Reference	Reference	Reference	Reference
	Yes	1.05 (0.43-2.56)	1.07 (0.44-2.61)	1.37 (0.53-3.53)	1.55 (0.72-3.37)
**Taking other medications currently**					
	No	Reference	Reference	Reference	Reference
	Yes	1.85 (0.77-4.45)	1.53 (0.63-3.71)	*4.23 (1.63-10.99)*	1.43 (0.65-3.17)
Medication safety perception		2.01 (0.64-6.36)	*3.37 (1.07-10.58)*	0.74 (0.2-2.68)	0.51 (0.17-1.47)
Perceived safety of communication with a physician		*3.59 (1.52-8.48)*	*2.44 (1.05-5.68)*	*9.06 (2.95-27.84)*	*2.68 (1.17-6.11)*
**Self-rated health**					
	Poor or fair	Reference	Reference	Reference	Reference
	Good, very good, or excellent	1.86 (0.76-4.58)	2.4 (0.99-5.78)	1.85 (0.7-4.86)	1.23 (0.58-2.62)
Patient activation		1.02 (0.58-1.81)	1.08 (0.61-1.92)	1.09 (0.6-1.99)	*1.83 (1.12-2.98)*
Medication self-management		0.91 (0.71-1.16)	1 (0.79-1.27)	1.02 (0.79-1.33)	1.19 (0.97-1.46)
**Experience of web-based self-reporting system**					
	No	Reference	Reference	Reference	Reference
	Yes	1.9 (0.76-4.75)	2.2 (0.88-5.48)	1.14 (0.44-2.93)	*3.8 (1.73-8.36)*
**Perceived importance of feedback**					
	Others	Reference	Reference	Reference	Reference
	Very important	*2.59 (1.03-6.51)*	2.5 (1-6.29)	*2.99 (1.11-8.05)*	*3.27 (1.37-7.78)*
**Influence of others’ self-reporting responses**					
	Not at all or do not know	Reference	Reference	Reference	Reference
	A lot or little	*2.53 (1.08-5.91)*	2.13 (0.92-4.92)	*3.36 (1.3-8.68)*	1.12 (0.55-2.3)

^a^OR: odds ratio.

^b^Italicized values denote the statistical significance of the *P* value (*P*<.05).

^c^OAA: oral anticancer agent.

## Discussion

### Principal Findings

This study demonstrated that patients with cancer had strong perceptions of medication and communication safety during transitions of care and a relatively high acceptance of digital technologies for self-reporting medication safety events after transitions back home. Furthermore, we identified significant factors associated with their technology acceptance, including patient activation, medication self-management ability, perceived medication and communication safety, perceived influence of seeing others’ self-reporting responses, and perceived importance of receiving feedback. Patients with cancer often experience transitions between different care settings, which place them at risk for adverse medication events [3,4]. A patient-oriented medication safety self-reporting system has the potential to engage patients and their families in self-reporting safety events from home settings, which can consequently enhance the understanding of the nature, scope, and causes of medication safety events occurring after patients transition back home and improve patient-centered cancer care [7,30-32]. The findings of this study increased the understanding of oncology patients’ willingness to adopt such a self-reporting system. This will contribute to the development of patient-facing technology systems tailored for self-reporting medication safety events. The associations between the factors, such as the perceived importance of feedback and the influence of others’ self-reporting responses, and technology acceptance (perceived usefulness, perceived ease of use, attitude toward use, and intention to use) could be further translated into system functionality and data representation, which are essential tasks for engaging patients.

As indicated in this study, oncology patients had a strong sense of medication safety and communication safety with their providers. These factors were positively associated with their activation level, ability to self-manage their medications, and acceptance of technology for self-reporting medication safety events, particularly their intention to use the self-reporting system. Patients’ concerns regarding their medication and communication safety were demonstrated to motivate their initiation and engagement in self-reporting medication safety events and their adoption of digital technology to improve medication safety. These findings are also perfectly aligned with the CCM, which emphasizes the effective interactions between patients who have been informed and activated and health care teams that are well-prepared and proactive, thus contributing to high-quality care outcomes [14,33]. Patient activation is not a new concept in oncology care settings. It has been reported to correlate with patients’ confidence in managing their OAA side effects and subsequent adherence [34]. Interpreted by cognitive-cultural conditions for patient willingness to share safety concerns, patients and families who are well equipped with adequate patient activation and abilities for medication self-management can understand and prioritize medication safety events and actively engage in self-reporting of medication safety events after they transition back home [7,35,36]. As an independent predictor of patients’ intention to use the self-reporting system, patient activation reflects their readiness and ability to be involved in their medication self-management. Therefore, to facilitate patient initiation and long-term engagement in using the medication safety event self-reporting system, the assessment and promotion of patient activation can be a fundamental design in developing the self-reporting system. It has been demonstrated that improving patient activation is feasible through continuous patient education, increased understanding of patient needs and expectations, and provision of personalized feedback and self-management recommendations [37,38]. In addition, digital technology as a tool, if designed and used appropriately, can significantly improve patients’ knowledge, skills, and confidence in self-management [36]. Therefore, a well-designed and developed patient-centered medication safety self-reporting system should be able to positively affect patient activation by providing accessible and useful medication information to support patient needs [39].

It is not surprising that prior technology use experience was a predictor of patients’ technology acceptance for self-reporting medication safety events, as theoretically, this factor is indicated in the Unified Technology Acceptance and Use Theory (UTAUT) [40]. Furthermore, it is understandable that patients’ perceptions of communication safety with health care providers can facilitate their intention to use the safety self-reporting system, which aligns with the interpersonal process of care and communication [41,42]. One notable finding was the association between patients’ perceptions of the importance of receiving their self-reporting feedback and their acceptance of the medication safety self-reporting system. Patients who preferred to receive feedback on their self-reporting were more likely to perceive the usefulness of the self-reporting system, have a positive attitude toward it, and have the intention to use it. This finding may confirm the prerequisite learning and change conditions from the literature, suggesting that a closed feedback loop between patients and clinicians is needed to improve patient safety [7,35,43]. Moreover, when patients and families serve as *vigilant partners* in medication safety self-monitoring and report their experiences and concerns after their care transitions back home, they can make a significant contribution to the understanding of the nature, scope, and causes of patient safety events outside health care systems that have been underreported historically [7,44,45].

To facilitate informed decision-making in cancer care, the medication safety self-reporting system should consider patient needs to enhance patient safety, promote self-management, and improve patient-centered care [46]. Patient-facing technologies, such as mobile health (mHealth) and wearable devices, are able to satisfy these expectations to increase the patient’s access to health information, support engagement in self-management, and improve communication with health care providers [47]. From the perspective of structural-procedural conditions for patient willingness to share safety concerns, patient-facing technologies are capable of generating personalized feedback based on the patient’s self-reporting and support interactive information exchanges to make the step-by-step process of self-reporting easy to follow, with downstream opportunities to increase patient engagement in the use of the system, improve patient outcomes, and reduce the cost of care in the long run [7,35,48,49]. Certainly, it is important to design these technologies to be user-friendly, secure, and accessible to all patients, especially to those who are at high risk of adverse medication events and in high need of self-management support, that is, patients who take OAAs and experience care transitions back home, being expected to manage their cancer treatments by themselves at home [50-53]. As reported by Beauchemin et al [53], the OAA adherence rates among these patients are suboptimal, ranging from 14% to 100%, depending on the agents and measures. Suboptimal OAA adherence can lead to poor survival, severe toxicities, and increased use of health care resources [54-56]. This study solicited perceptions, willingness, and acceptance of technologies to support the subsequent development of effective programs for patient-centered and evidence-based cancer care.

### Comparison With Prior Work

Constructs from the CCM, Technology Acceptance Model (TAM), and UTAUT were adopted in this study to help understand oncology patients’ acceptance and use of digital technologies for self-reporting medication safety events after their care transitions back home [14,40]. Previous studies have explored the acceptance and use of mHealth or eHealth apps for self-management among the survivors of cancer and revealed whether personal factors, such as age, education, marital status, and employment status, and clinical factors, such as cancer diagnosis timeframe and that survivors are undergoing active treatment, mattered or not [22]. Consistently, this study suggested similar personal factors, including education, marital status, and employment status, for technology acceptance. However, since the previous study by de Brun et al [13] focused on general patient self-management instead of the self-reporting of medication safety events specifically, it was not able to address one of the main constructs of the CCM, which is a facilitator of involving the informed and activated persons in the process of productive interactions with health care teams, as indicated in this study [14]. Another study by Jiang et al [22] was a systematic review of the acceptance and use of home-based electronic symptom self-reporting systems by patients with cancer. This review is also guided by the CCM and UTAUT and has suggested that the interactive system features can improve patients’ engagement in self-reporting, which is congruent with this study [23]. Furthermore, this review criticizes that existing home-based symptom self-reporting systems lack personalization features and only use questionnaires to collect patients’ self-reporting data, which could not meet patients’ various needs for self-reporting their safety concerns more conveniently and flexibly at any time [23]. This study had a similar finding, highlighting the patients’ strong concerns regarding medication safety. It indicated that patients with cancer want to receive feedback on what they have reported at a personalized level. Although the TAM and UTAUT has been widely used to identify factors associated with technology system acceptance and use, its implications for the medical domain have been frequently criticized for lacking consideration of the complex health care context and need to be reassessed for additional and external factors that match with the targeted health context [57,58]. The implication of TAM and UTAUT in the context of self-reporting medication safety events in patients with cancer has not been reported before. Self-reporting of medication safety events includes but is not limited to self-reporting of symptoms (or adverse effects), medication nonadherence, medication self-administration errors, drug-drug or drug-food interactions, and safe handling or storage of OAAs. Overall, no previous study has targeted technology acceptance for self-reporting of medication safety events after oncology patients’ care transitions back home [59].

### Limitations

This study had several limitations. First, with a cross-sectional design, the study could not follow up with patients to understand the changes in their perception over time. It also could not demonstrate the causal relationships between the identified factors and technology acceptance (perceived usefulness, perceived ease of use, attitude toward use, and intention to use). However, the sample size of 204 participants had adequate power to reveal the potential associations among them. Furthermore, theoretical frameworks, such as the CCM, TAM, and UTAUT, were used to guide the identification and interpretation of potential factors. Second, the study was conducted at a single site, which had the potential limitation of reaching a homogenous group. Therefore, the findings may not be generalizable to other settings or regions. Third, no existing medication safety self-reporting system can be provided to assess patients’ actual technology use experience. Although a scenario that describes the possible system was demonstrated in the survey, some participants may have had difficulty envisioning a pseudosystem unless they have actually used it. Therefore, longitudinal studies are needed to explore patients’ acceptance and actual use of digital technologies for self-reporting medication safety events to completely understand patients’ long-term engagement behaviors.

### Conclusions

This study provides new insights into oncology patients’ perceptions of safety during care transitions and their willingness to use digital technologies to self-report medication safety events after care transitions back home. Specifically, it highlights the importance of improving patient activation and medication self-management abilities to potentially increase their understanding and capabilities to prioritize medication safety events and engage them in using digital technology for self-reporting medication safety events. In addition, improving patients’ technology use experience through appropriate training programs; assessing patients’ perceptions of safety communication with health care providers; and integrating personalized features in the system design, such as providing individualized feedback on patient self-reporting, should be able to facilitate technology acceptance for self-reporting medication safety events in patients with cancer. As the informed and activated patients are more likely to have the knowledge and capability to initiate and engage in self-reporting, developing a patient-centered reporting system to empower patients and their families and facilitate safety health communications will help oncology patients in addressing their medication safety concerns, meeting their care needs, and holding promise to improve the quality of cancer care.

## Appendix

Multimedia Appendix 1 STROBE (Strengthening the Reporting of Observational Studies in Epidemiology) guidelines.

Multimedia Appendix 2 International Classification of Diseases, Tenth Revision codes for the 4 eligible cancer types.

Multimedia Appendix 3 Survey questionnaires.

## Abbreviations

CCM: Chronic Care Model
mHealth: mobile health
OAA: oral anticancer agent
OR: odds ratio
STROBE: Strengthening the Reporting of Observational Studies in Epidemiology
TAM: Technology Acceptance Model
UTAUT: Unified Technology Acceptance and Use Theory

## Data Availability

The data supporting the findings of this study are available from the corresponding author upon reasonable request.
