# Supplementary material for: The Acceptance and Use of Digital Technologies for Self-Reporting Medication Safety Events After Care Transitions to Home in Patients With Cancer: Survey Study
Source: J Med Internet Res. 2024 Mar 8;26:e47685. doi: 10.2196/47685 (PMC10960221; doi:10.2196/47685)
Supplement: Multimedia Appendix 2 [file jmir_v26i1e47685_app2.docx]

| **Eligible diagnosis** | **ICD-10 code** |
| --- | --- |
| Malignant neoplasm of colon | C18; C18.0-18.9 |
| Malignant neoplasm of rectosigmoid junction | C19 |
| Malignant neoplasm of rectum | C20 |
| Malignant neoplasm of bronchus or lung | C34.1; C34.10-34.12; C34.2, C34.8; C34.80-34.81; C34.9, C34.90-34.92 |
| Malignant neoplasm of breast | C50; C50.0-C50.6; C50.8-50.9; C50.01-50.02; C50.11-50.12; C50.21-50.22; C50.31-50.32; C50.41-50.42; C50.51-50.52; C50.61-50.62; C50.81-50.82; C50.91-50.92; C50.011-50.012; C50.019; C50.021-50.022; C50.029; C50.111-50.112; C50.119; C50.121-50.122; C50.129; C50.211-50.212; C50.219; C50.221-50.222; C50.229; C50.311-50.312; C50.319; C50.321-50.322; C50.329; C50.411-50.412; C50.419; C50.421-50.422; C50.429; C50.511-50.512; C50.519; C50.521-50.522; C50.529; C50.611-50.612; C50.619; C50.621-50.622; C60.529; C50.811-50.812; C50.819; C50.821-50.822; C50.829; C50.911-50.912; C50.919; C50.921-50.922; C50.929 |
| Malignant neoplasm of prostate | C61 |
