# Supplementary material for: The Acceptance and Use of Digital Technologies for Self-Reporting Medication Safety Events After Care Transitions to Home in Patients With Cancer: Survey Study
Source: J Med Internet Res. 2024 Mar 8;26:e47685. doi: 10.2196/47685 (PMC10960221; doi:10.2196/47685)
Supplement: Multimedia Appendix 3 [file jmir_v26i1e47685_app3.pdf]

Medication Safety Event Reporting Survey

Section 1: Demographics

1. What is your age?

_________ years old.

2. What is your gender?

☐ Female
☐ Male
☐ Prefer to self-describe ______________________
☐ Prefer not to answer

3. What is your marital status?

☐ Currently married or living as married
☐ Currently unmarried

4. Are you of Hispanic or Latino/a, that is, of Mexican, Puerto Rican, Cuban, Caribbean, or of Latin American descent?

☐ Yes
☐ No
☐ Do Not Know

5. What is your race?

☐ American Indian/Alaska Native
☐ Asian
☐ Native Hawaiian or Other Pacific Islander
☐ Black or African American
☐ White
☐ More than One Race

6. How well do you speak English?

☐ Very well
☐ Well

☐ Not well

7. What is your educational background?

☐ Grade school (Grades 1-8)
☐ High school (Grades 9-12)
☐ Some college
☐ College graduate
☐ Postgraduate

8. What is your current occupational status?

☐ Full time
☐ Part time
☐ Unemployed
☐ Homemaker
☐ Student
☐ Retired
☐ Disabled
☐ Other – Specify: _______________

9. Does your current household income meet your basic needs (such as food, housing, utilities, and healthcare)?

☐ Yes
☐ No

Section 2: Your Overall Health

1. In general, would you say your health is:

Excellent	Very Good	Good	Fair	Poor
○	○	○	○	○

2. Overall, how confident are you about your ability to take good care of your health?

Completely Confident	Very Confident	Somewhat Confident	A Little Confident	Not Confident at all
○	○	○	○	○

3. Do you have friends or family members that you talk to about your health?

☐ Yes
☐ No

4. Some people avoid visiting their doctor even when they suspect they should. Would you say this is true for you, or not true for you?

☐ True
☐ Not true

Section 4: Beliefs about Medications

1. My health, at present, depends on my medications.

Strongly Disagree	Disagree	Uncertain	Agree	Strongly Agree
○	○	○	○	○

2. Having to take medications worries me.

Strongly Disagree	Disagree	Uncertain	Agree	Strongly Agree
○	○	○	○	○

3. My life would be impossible without my medications.

Strongly Disagree	Disagree	Uncertain	Agree	Strongly Agree
○	○	○	○	○

4. Without my medications, I would be very ill.

Strongly Disagree	Disagree	Uncertain	Agree	Strongly Agree
○	○	○	○	○

5. I sometimes worry about the long-term effects of my medications.

Strongly Disagree	Disagree	Uncertain	Agree	Strongly Agree
○	○	○	○	○

6. My medications are a mystery to me.

Strongly Disagree	Disagree	Uncertain	Agree	Strongly Agree
○	○	○	○	○

7. My medications disrupt my life.

Strongly Disagree	Disagree	Uncertain	Agree	Strongly Agree
○	○	○	○	○

8. My health in the future will depend on my medications.

Strongly Disagree	Disagree	Uncertain	Agree	Strongly Agree
○	○	○	○	○

9. My medications protect me from becoming worse.

Strongly Disagree	Disagree	Uncertain	Agree	Strongly Agree
○	○	○	○	○

10. I sometimes worry about becoming too dependent on my medications.

Strongly Disagree	Disagree	Uncertain	Agree	Strongly Agree
○	○	○	○	○

Section 5: Medication Safety Perception

1. **How safe did you feel about your medication during your transitions of care from the hospital or clinic to home last time?**
   (For example, receiving the correct medication, understanding the medication you were taking, or delays in receiving your medication)

Completely Safe	Safe	Uncertain	Unsafe	Completely Unsafe
○	○	○	○	○

2. How safe do you feel about taking your medication at home?

(For example, knowing when to take, how frequent to take, how many to take each time, and how to take, for example, with or without food)

Completely Safe	Safe	Uncertain	Unsafe	Completely Unsafe
○	○	○	○	○

3. How safe did you feel about handling any possible side effects of your medication at home?

(For example, if you felt confident that you were aware of possible side effects of your medication and know how to track or deal with those side effects)

Completely Safe	Safe	Uncertain	Unsafe	Completely Unsafe
○	○	○	○	○

4. Overall, how safe did you feel throughout the whole transfer from the hospital or clinic to home?

Completely Safe	Safe	Uncertain	Unsafe	Completely Unsafe
○	○	○	○	○

Section 6: Safe of Communication

1. How safe did the communication from clinicians make you feel? (For example, giving you clear and timely information or being polite)

Completely Safe	Safe	Uncertain	Unsafe	Completely Unsafe
○	○	○	○	○

2. How safe did you feel with regards to clinicians listening to you and responding to your individual needs?

Completely Safe	Safe	Uncertain	Unsafe	Completely Unsafe
○	○	○	○	○

Section 7: Patient Activation Measure

Please indicate if you would agree or disagree with the following statements.

1. When all is said and done, I am the person who is responsible for managing my health condition.

Strongly Disagree	Disagree	Uncertain	Agree	Strongly Agree
○	○	○	○	○

2. Taking an active role in my own health care is the most important factor in determining my health and ability to function.

Strongly Disagree	Disagree	Uncertain	Agree	Strongly Agree

○	○	○	○	○

3. I am confident that I can take actions that will help prevent or minimize some symptoms or problems associated with my health condition.

Strongly Disagree	Disagree	Uncertain	Agree	Strongly Agree
○	○	○	○	○

4. I know what each of my prescribed medications do.

Strongly Disagree	Disagree	Uncertain	Agree	Strongly Agree
○	○	○	○	○

5. I am confident that I can tell when I need to go get medical care and when I can handle a health problem myself.

Strongly Disagree	Disagree	Uncertain	Agree	Strongly Agree
○	○	○	○	○

6. I am confident I can tell my healthcare provider concerns I have even when he or she does not ask.

Strongly Disagree	Disagree	Uncertain	Agree	Strongly Agree
○	○	○	○	○

7. I am confident that I can follow through on medical treatments I need to do at home.

Strongly Disagree	Disagree	Uncertain	Agree	Strongly Agree
○	○	○	○	○

8. I understand the nature and causes of my health condition(s).

Strongly Disagree	Disagree	Uncertain	Agree	Strongly Agree
○	○	○	○	○

9. I know the different medical treatment options available for my health condition.

Strongly Disagree	Disagree	Uncertain	Agree	Strongly Agree
○	○	○	○	○

10. I have been able to maintain the lifestyle changes for my health that I have made.

Strongly Disagree	Disagree	Uncertain	Agree	Strongly Agree
○	○	○	○	○

11. I know how to prevent further problems with my health condition.

Strongly Disagree	Disagree	Uncertain	Agree	Strongly Agree
○	○	○	○	○

12. I am confident I can figure out solutions when new situations or problems arise with my health condition.

Strongly Disagree	Disagree	Uncertain	Agree	Strongly Agree
○	○	○	○	○

13. I am confident that I can maintain lifestyle changes like diet and exercise even during times of stress.

Strongly Disagree	Disagree	Uncertain	Agree	Strongly Agree
○	○	○	○	○

Section 8: Technology Acceptance and Use

1. In the last 12 months, have you used any electronic devices (e.g. computer, mobile phone) to report, monitor, or track your health?

☐ Yes
☐ No
☐ I do not have access to those devices

2. In the past 12 months, have you used e-mail or the Internet to communicate with a doctor or doctor's office; or request prescription renewals?

☐ Yes
☐ No
☐ I do not have access to the Internet

3. In the past 12 months, have you used any of the following to report and exchange medical information with a healthcare professional? (Select all that applies)

- ☐ E-mail
- ☐ Text message
- ☐ App on a smartphone or mobile device
- ☐ Video conference (e.g., Zoom, Facetime, etc.)
- ☐ Social media
- ☐ Fax
- ☐ None of the above

4. Have you used any online Patient Portal (such as MyUofMHealth)?

- ☐ Yes
- ☐ No

5. Have you reported any medication-related safety issues or concerns using an online reporting system?

- ☐ Yes (If Yes: Answer Q6 below)
- ☐ No

6. Who reported your health status, medication-related safety issues, or concerns in the online reporting system?

- □ By yourself
- □ Caregiver
- □ Others (Please specify): _________

Scenario: Pretend there is an online system in your computer or mobile device. You can use it to report any unsafe experiences or concerns about your medications at any time. This online system can automatically provide self-care recommendations. Health professional can use it to follow up with you for your reporting. You can also get a summary of reports from you and other people who have had similar care processes like you.

7. How likely would you consider this online reporting system is useful?

Very Likely	Likely	Uncertain	Unlikely	Very Unlikely
○	○	○	○	○

8. How likely would you consider it is easy to use such an online reporting system?

Very Likely	Likely	Uncertain	Unlikely	Very Unlikely
○	○	○	○	○

9. How would you think about reporting your safety experiences or concerns through such an online reporting system?

Very Positive	Positive	Neither	Negative	Very Negative
○	○	○	○	○

10. Do you intend to use an online reporting system to report your safety concerns?

Very Likely	Likely	Uncertain	Unlikely	Very Unlikely
○	○	○	○	○

11. How likely do you think seeing others' reporting responses and examples can influence whether or not you will report certain information?

Very Likely	Likely	Uncertain	Unlikely	Very Unlikely
○	○	○	○	○

Section 9: Medication Event Reporting Feedback

1. How important does the feedback to your reporting encourage you to continue reporting your experiences or concerns of safety issues?

Extremely Important	Very	Somewhat	A Little	Not Important at all	No Previous Experience
○	○	○	○	○	○

2. How soon do you expect to receive the feedback to your reporting of safety issues?

- ☐ In a few hours
- ☐ On the same day
- ☐ On the next day
- ☐ In a couple of days
- ☐ More than two days

3. If the online reporting system can immediately provide self-care recommendations, how likely would you accept the feedback from the system?

Very Likely	Likely	Uncertain	Unlikely	Very Unlikely
○	○	○	○	○

Section 10: Medication Self-Management

Directions: we would like to ask you a few questions to make sure you are taking your medication safely.

1. Did you forget to take your medications at any time last week?

- ☐ Yes

☐ No
☐ I don’t know

2. In the past month, have you stopped taking your medications for any reason without telling your doctor?

☐ Yes
☐ No
☐ I don’t know

For the next set of questions, please tell us how often the following statements are true for you.

3. I often forget to take my medications.

Never	Some of the Time	Most of the Time	All of the Time
○	○	○	○

4. I am organized about when and how I take my medications.

Never	Some of the Time	Most of the Time	All of the Time
○	○	○	○

5. I have a hard time paying for my medications.

Never	Some of the Time	Most of the Time	All of the Time
○	○	○	○

For the last set of questions, please tell us if you agree or disagree with the following statements.

6. The print instructions on my prescription bottles are confusing.

Agree	Disagree	Uncertain
○	○	○

7. I often have a hard time remembering if I have already taken my medications.

Agree	Disagree	Uncertain
○	○	○

8. I do not take my medications when I am feeling sad or upset.

Agree	Disagree	Uncertain
○	○	○

9. When my medication causes minor side effects, I stop taking it.

Agree	Disagree	Uncertain
○	○	○

10. The idea of taking medications for the rest of my life makes me very uncomfortable.

Agree	Disagree	Uncertain
○	○	○
